# Prognostic value of Mandard score and nodal status for recurrence patterns and survival after multimodal treatment of oesophageal adenocarcinoma

**DOI:** 10.1093/bjs/znae034

**Published:** 2024-02-22

**Authors:** Sofie P G Henckens, Dajia Liu, Suzanne S Gisbertz, Marianne C Kalff, Maarten C J Anderegg, David Crull, Freek Daams, Annette D van Dalsen, Jan Willem T Dekker, Marc J van Det, Peter van Duijvendijk, Wietse J Eshuis, Richard P R Groenendijk, Jan Willem Haveman, Richard van Hillegersberg, Misha D P Luyer, Pim B Olthof, Jean-Pierre E N Pierie, Victor D Plat, Camiel Rosman, Jelle P Ruurda, Johanna W van Sandick, Meindert N Sosef, Daan M Voeten, Guy H E J Vijgen, Maarten F Bijlsma, Sybren L Meijer, Maarten C C M Hulshof, Cesar Oyarce, Sjoerd M Lagarde, Hanneke W M van Laarhoven, Mark I van Berge Henegouwen, IVORY study group, Peter C Baas, Renu R Bahadoer, Eric J T Belt, Baukje Brattinga, Linda Claassen, Admira Ćosović, Manon Drost, Stijn van Esser, Marcia P Gaspersz, Burak Görgec, Henk H Hartgrink, Erwin van der Harst, Joos Heisterkamp, Wendy Kelder, B Feike Kingma, Willem J Koemans, Ewout A Kouwenhoven, Frederik Lecot, Philip P van der Linden, Grard A P Nieuwenhuijzen, Martijn G H van Oijen, Donald L van der Peet, E G J M Robert Pierik, Fatih Polat, Rene Scheer, Cettela A M Slootmans, Odin V Sosef, Wobbe O de Steur, Hein B A C Stockmann, Fanny J Stoop, Guusje Vugts, Víola B Weeda, Marinus J Wiezer

**Affiliations:** Department of Surgery, Amsterdam UMC, Location University of Amsterdam, Amsterdam, the Netherlands; Cancer Centre Amsterdam, Cancer Treatment and Quality of Life, Amsterdam, the Netherlands; Department of Gastroenterology and Hepatology, Amsterdam UMC, Location University of Amsterdam, Amsterdam Gastroenterology Endocrinology Metabolism, Amsterdam, the Netherlands; Department of Medical Oncology, Amsterdam UMC, Location University of Amsterdam, Amsterdam, the Netherlands; Centre for Experimental and Molecular Medicine, Laboratory for Experimental Oncology and Radiobiology, Amsterdam UMC, Location University of Amsterdam, Amsterdam, the Netherlands; Department of Surgery, Amsterdam UMC, Location University of Amsterdam, Amsterdam, the Netherlands; Cancer Centre Amsterdam, Cancer Treatment and Quality of Life, Amsterdam, the Netherlands; Department of Gastroenterology and Hepatology, Amsterdam UMC, Location University of Amsterdam, Amsterdam Gastroenterology Endocrinology Metabolism, Amsterdam, the Netherlands; Department of Surgery, Amsterdam UMC, Location University of Amsterdam, Amsterdam, the Netherlands; Cancer Centre Amsterdam, Cancer Treatment and Quality of Life, Amsterdam, the Netherlands; Department of Gastroenterology and Hepatology, Amsterdam UMC, Location University of Amsterdam, Amsterdam Gastroenterology Endocrinology Metabolism, Amsterdam, the Netherlands; Department of Surgery, Amsterdam UMC, Location University of Amsterdam, Amsterdam, the Netherlands; Cancer Centre Amsterdam, Cancer Treatment and Quality of Life, Amsterdam, the Netherlands; Department of Gastroenterology and Hepatology, Amsterdam UMC, Location University of Amsterdam, Amsterdam Gastroenterology Endocrinology Metabolism, Amsterdam, the Netherlands; Department of Surgery, Ziekenhuisgroep Twente, Almelo, the Netherlands; Department of Surgery, Amsterdam UMC, Location Vrije Universiteit Amsterdam, Amsterdam, the Netherlands; Department of Surgery, Isala Klinieken, Zwolle, the Netherlands; Department of Surgery, Reinier de Graaf Groep, Delft, the Netherlands; Department of Surgery, Ziekenhuisgroep Twente, Almelo, the Netherlands; Department of Surgery, Gelre Ziekenhuis, Apeldoorn, the Netherlands; Department of Surgery, Amsterdam UMC, Location University of Amsterdam, Amsterdam, the Netherlands; Cancer Centre Amsterdam, Cancer Treatment and Quality of Life, Amsterdam, the Netherlands; Department of Gastroenterology and Hepatology, Amsterdam UMC, Location University of Amsterdam, Amsterdam Gastroenterology Endocrinology Metabolism, Amsterdam, the Netherlands; Department of Surgery, IJsselland Ziekenhuis, Capelle aan den IJssel, the Netherlands; Department of Surgery, University Medical Centre Groningen, University of Groningen, Groningen, the Netherlands; Department of Surgery, UMC Utrecht, Utrecht, the Netherlands; Department of Surgery, Catharina Ziekenhuis, Eindhoven, the Netherlands; Department of Surgery, Reinier de Graaf Groep, Delft, the Netherlands; Department of Surgery, MC Leeuwarden, Leeuwarden, the Netherlands; Department of Surgery, Amsterdam UMC, Location Vrije Universiteit Amsterdam, Amsterdam, the Netherlands; Department of Surgery, Radboud University Medical Centre, Nijmegen, the Netherlands; Department of Surgery, UMC Utrecht, Utrecht, the Netherlands; Department of Surgery, Antoni van Leeuwenhoek Ziekenhuis, Amsterdam, the Netherlands; Department of Surgery, Zuyderland, Heerlen, the Netherlands; Department of Surgery, Amsterdam UMC, Location University of Amsterdam, Amsterdam, the Netherlands; Cancer Centre Amsterdam, Cancer Treatment and Quality of Life, Amsterdam, the Netherlands; Department of Gastroenterology and Hepatology, Amsterdam UMC, Location University of Amsterdam, Amsterdam Gastroenterology Endocrinology Metabolism, Amsterdam, the Netherlands; Department of Surgery, Erasmus Medical Centre, Rotterdam, the Netherlands; Cancer Centre Amsterdam, Cancer Treatment and Quality of Life, Amsterdam, the Netherlands; Centre for Experimental and Molecular Medicine, Laboratory for Experimental Oncology and Radiobiology, Amsterdam UMC, Location University of Amsterdam, Amsterdam, the Netherlands; Oncode Institute, Amsterdam, the Netherlands; Cancer Centre Amsterdam, Cancer Treatment and Quality of Life, Amsterdam, the Netherlands; Department of Pathology, Amsterdam UMC, Location University of Amsterdam, Amsterdam, the Netherlands; Cancer Centre Amsterdam, Cancer Treatment and Quality of Life, Amsterdam, the Netherlands; Department of Radiotherapy, Amsterdam UMC, Location University of Amsterdam, Amsterdam, the Netherlands; Centre for Experimental and Molecular Medicine, Laboratory for Experimental Oncology and Radiobiology, Amsterdam UMC, Location University of Amsterdam, Amsterdam, the Netherlands; Oncode Institute, Amsterdam, the Netherlands; Department of Surgery, Erasmus Medical Centre, Rotterdam, the Netherlands; Cancer Centre Amsterdam, Cancer Treatment and Quality of Life, Amsterdam, the Netherlands; Department of Medical Oncology, Amsterdam UMC, Location University of Amsterdam, Amsterdam, the Netherlands; Department of Surgery, Amsterdam UMC, Location University of Amsterdam, Amsterdam, the Netherlands; Cancer Centre Amsterdam, Cancer Treatment and Quality of Life, Amsterdam, the Netherlands; Department of Gastroenterology and Hepatology, Amsterdam UMC, Location University of Amsterdam, Amsterdam Gastroenterology Endocrinology Metabolism, Amsterdam, the Netherlands

## Abstract

**Background:**

This study evaluated the association of pathological tumour response (tumour regression grade, TRG) and a novel scoring system, combining both TRG and nodal status (TRG-ypN score; TRG1-ypN0, TRG>1-ypN0, TRG1-ypN+ and TRG>1-ypN+), with recurrence patterns and survival after multimodal treatment of oesophageal adenocarcinoma.

**Methods:**

This Dutch nationwide cohort study included patients treated with neoadjuvant chemoradiotherapy followed by oesophagectomy for distal oesophageal or gastro-oesophageal junctional adenocarcinoma between 2007 and 2016. The primary endpoint was the association of Mandard score and TRG-ypN score with recurrence patterns (rate, location, and time to recurrence). The secondary endpoint was overall survival.

**Results:**

Among 2746 inclusions, recurrence rates increased with higher Mandard scores (TRG1 30.6%, TRG2 44.9%, TRG3 52.9%, TRG4 61.4%, TRG5 58.2%; *P* < 0.001). Among patients with recurrent disease, the distribution (locoregional *versus* distant) was the same for the different TRG groups. Patients with TRG1 developed more brain recurrences (17.7 *versus* 9.8%; *P* = 0.001) and had a longer mean overall survival (44 *versus* 35 months; *P* < 0.001) than those with TRG>1. The TRG>1-ypN+ group had the highest recurrence rate (64.9%) and worst overall survival (mean 27 months). Compared with the TRG>1-ypN0 group, patients with TRG1-ypN+ had a higher risk of recurrence (51.9 *versus* 39.6%; *P* < 0.001) and worse mean overall survival (33 *versus* 41 months; *P* < 0.001).

**Conclusion:**

Improved tumour response to neoadjuvant therapy was associated with lower recurrence rates and higher overall survival rates. Among patients with recurrent disease, TRG1 was associated with a higher incidence of brain recurrence than TRG>1. Residual nodal disease influenced prognosis more negatively than residual disease at the primary tumour site.

## Teaser

In this Dutch nationwide cohort study, improved tumour response to neoadjuvant therapy was associated with a lower recurrence rate and higher overall survival rate. Among patients with recurrent disease, tumour regression grade (TRG) 1 was associated with a higher incidence of brain recurrences than TRG greater than 1. Residual nodal disease influenced prognosis more negatively than residual disease at the primary tumour site.

## Introduction

Since demonstrating a survival benefit in the large randomized controlled CROSS trial, the curative treatment strategy for patients with resectable oesophageal cancer without distant metastases has consisted of neoadjuvant chemo(radio)therapy followed by oesophagectomy^1–4^. After neoadjuvant chemoradiotherapy and surgery, the 5-year survival rate is around 50%^5–7^. This modest survival can be explained by therapeutic resistance, early dissemination, and disease recurrence^8,9^. The system used most widely to evaluate response to neoadjuvant therapy is the Mandard tumour regression grade (TRG), which describes the proportion of primary tumour mass in the resection specimen replaced by fibrosis following neoadjuvant systemic and/or local treatment^10^. This ratio is translated into a five-point scale from TRG1 (complete response) to TRG5 (absence of response). Response to neoadjuvant therapy is associated with prognosis, with superior survival for patients with complete tumour regression^10–13^. The pCR rate among patients with oesophageal cancer varies from 20 to 50%, depending on, among others, histological tumour type^2,14^.

Recurrent oesophageal cancer develops in approximately half of patients after treatment with curative intent^9,15^. It is not fully understood how response to neoadjuvant therapy is associated with patterns of recurrent disease, although this may be relevant to surveillance and treatment of postoperative recurrences.

Besides the Mandard score, which is solely based on residual tumour mass at the primary tumour site in the oesophagus or at the gastro-oesophageal junction, there are several other important determinants of prognosis. Previous studies have shown that pathological lymph node status after neoadjuvant chemoradiotherapy (ypN) is independently associated with prognosis and does not always correlate with response at the primary tumour site^16–19^. Using a novel four-tier scoring system, in which both treatment response at the primary oesophageal tumour site (TRG1 *versus* TRG>1) and nodal status (ypN0 *versus* ypN+) are combined (TRG-ypN score), could lead to enhanced prognostic accuracy. The present study aimed to evaluate the prognostic value of the Mandard score and TRG-ypN score for patterns of recurrent disease and survival of patients with oesophageal adenocarcinoma.

## Methods

### Study design

This study was a *post hoc* analysis of the Dutch nationwide IVORY study, which evaluated the patterns of surgical care for distal oesophageal and gastro-oesophageal junctional cancer^20^. The IVORY study included all patients who underwent oesophageal cancer surgery in the Netherlands between January 2007 and December 2016. Approval for the IVORY study was obtained from the institutional review board of each participating centre. The STROBE guidelines for observational studies were used to ensure correct reporting of study results^21^.

### Patients

Patients who underwent multimodal treatment, consisting of neoadjuvant chemoradiotherapy and oesophagectomy with gastric conduit reconstruction, for a primary adenocarcinoma of the distal oesophagus or gastro-oesophageal junction between 2007 and 2016 were included in the present study. Patients for whom tumour regression or recurrence status was not documented were excluded.

### Outcomes

The primary endpoint was recurrence pattern including recurrence rate, location of recurrent disease, and time to recurrence. The secondary endpoint was overall survival (OS). Follow-up data on disease recurrence and survival status were collected until January 2020^20^.

### Treatment and follow-up

Neoadjuvant chemoradiotherapy was administered to all patients included in this study. According to the Dutch national guidelines, this was mostly according to the CROSS regimen (23 fractions of 1.8 Gy (41.4 Gy) conformal external-beam radiotherapy combined with cycles of carboplatin administered 5 weekly (area under the curve 2 mg per ml per min) and paclitaxel (50 mg/m^2^ for 23 days))^2^. Oesophagectomy was performed through an open, minimally invasive, or hybrid transthoracic or transhiatal approach^22,23^.

In accordance with the Dutch national guidelines, follow-up outpatient visits were planned at intervals of 3 months during the first postoperative year, every 6 months during years 2–4, and once more during year 5 after surgery. No routine radiological or endoscopic follow-up was conducted, and follow-up consisted of medical history and physical examination. When recurrent disease was suspected or symptoms occurred, easily accessible (PET–)CT and/or endoscopy with biopsies was carried out.

### Pathology

Pathology reports included tumour histology, resection margin status, and the number and aspect of resected lymph nodes. To grade response to neoadjuvant chemoradiotherapy, the degree of histomorphological regression was classified using the Mandard score. Generally, lymph nodes were embedded in total and routinely processed before haematoxylin and eosin staining was performed to assess pathological lymph node status. If indicated, additional CAM 5.2 immunohistochemical staining techniques were used to detect individual vital tumour cells (isolated tumour cells) or micrometastases. Pathological staging was determined using the AJCC/UICC classification of malignant tumours of the oesophagus and oesophagogastric junction^24,25^.

### Definitions

**Fig. 1:**
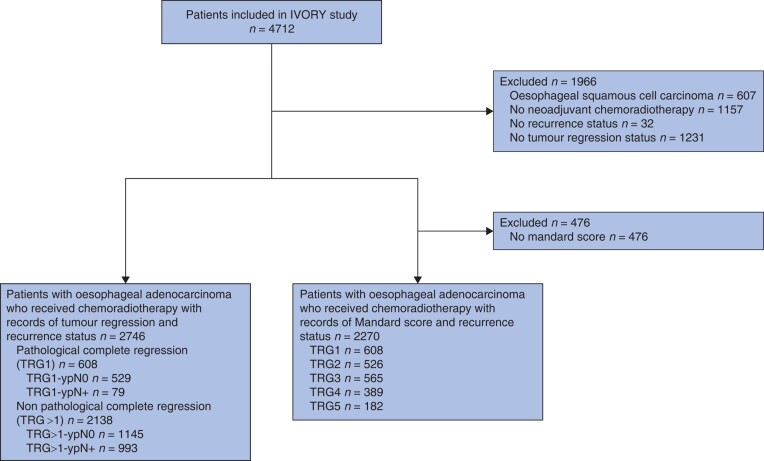
Study flow chart

Location of disease recurrence was classified as locoregional only (close to the initial tumour site or in locoregional lymph nodes), distant only (in distant organs or non-regional lymph nodes), or combined (co-existing at locoregional and distant sites, regardless of the timing of occurrence). OS was defined as the interval from date of surgery to date of death or last follow-up. The Mandard TRG was used to evaluate the response to neoadjuvant therapy. This grade describes the proportion of primary tumour mass in the resection specimen that is replaced by fibrosis after neoadjuvant treatment. It is graded on a five-point scale from TRG1 (complete response: 100% fibrosis, no viable tumour cells) to TRG5 (absence of response: no fibrosis, 100% viable tumour cells)^10^. A comparison of response to neoadjuvant therapy was done for both the five-tier system (TRG1, TRG2, TRG3, TRG4, and TRG5), as well as for two groups (TRG1 and TRG>1). Some 476 patients with partial tumour regression, but missing a specific Mandard score, were included in the group with TRG>1. For the TRG-ypN analyses, in which treatment response at the primary tumour site and pathological nodal status (ypN) were combined, a novel four-tier system was created: TRG1-ypN0, TRG>1-ypN0, TRG1-ypN+, and TRG>1-ypN+ (*Fig. 1*).

Among all 4712 patients from the IVORY study, all 2746 with oesophageal adenocarcinoma who received neoadjuvant chemoradiotherapy and underwent oesophagectomy, with records of tumour regression and recurrence status, were included. The 476 patients with partial tumour regression, but missing specific Mandard scores, were included in the group of patients with tumour regression grade (TRG) exceeding 1, but not in the analyses of 2270 patients stratified by specific Mandard scores. The sum of exclusions may not add up to the total number of excluded patients, as multiple reasons may apply to one patient.

### Statistical analysis

Outcomes are reported as mean(s.d.) for normally distributed variables, median (i.q.r.) for non-normally distributed variables, and numbers with percentages for categorical variables. Variables were compared using independent *t*, Mann–Whitney *U* or χ^2^ tests, as appropriate Survival curves were estimated using the Kaplan–Meier method and compared using log rank tests. When survival probability did not reach a minimum of 50% for each group, mean survival times were calculated instead of median values. Statistical analyses were conducted with SPSS^®^ version 28.0 (IBM, Armonk, NY, USA). For all analyses, two-sided *P* < 0.050 was considered statistically significant.

## Results

### Study population

**Fig. 2:**
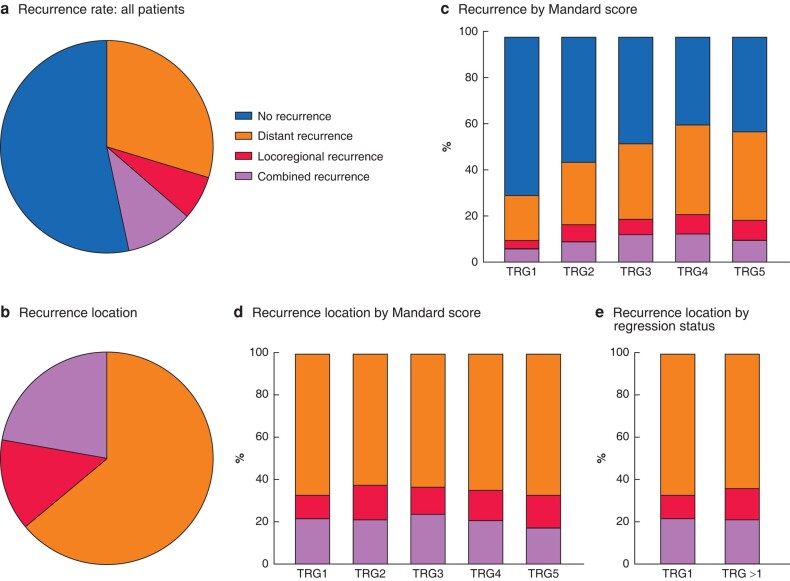
Recurrence patterns, stratified by Mandard score

Of all 4712 patients included in the IVORY study, a total of 2746 with oesophageal adenocarcinoma were treated with neoadjuvant chemoradiotherapy followed by surgical resection, and therefore included in the present study (*Fig. 1*). Patients were predominantly men (84.4%) with a mean(s.d.) age of 64.2(9.0) years. The majority was diagnosed with a tumour in the distal oesophagus (76.7%). A transthoracic resection was performed in 1831 patients (66.7%), and the remaining 915 (33.3%) had a transhiatal oesophagectomy (*Table 1*). The resection was complete (R0) in 2633 patients (96.1%) and the median lymph node yield was 18 (i.q.r. 13–24). Median lymph node yield was 13 (9–18) for patients who had a transhiatal procedure *versus* 20 (15–26) for those who had transthoracic surgery (*P* < 0.001). A complete response (TRG1) was observed in 608 patients (26.8%); TRG2 occurred in 526 (23.2%), TRG3 in 565 (24.9%), TRG4 in 389 (17.1%), and TRG5 in 182 (8.0%) (*Fig. 1*). During follow-up, recurrent disease was diagnosed in 1283 patients (46.7%); it was locoregional in 6.5%, distant in 30.0%, and combined in 10.3% (*Fig. 2a*). Among patients with recurrence, 13.6% had locoregional recurrence only, 64.2% distant only, and 22.2% combined recurrence (*Fig. 2b*).

**a** Recurrence rate among all 2746 patients. **b** Recurrence location distribution pattern in 1066 patients with recurrence. **c** Recurrence rate by Mandard score among all 2270 patients with recorded Mandard score. **d** Recurrence location distribution pattern by Mandard score among 1056 patients with recurrence. **e** Recurrence location distribution pattern by regression status among 1066 patients with recurrence. **c***P* < 0.001, **d***P* = 0.797, **e***P* = 0.491 (χ^2^ test).

**Table 1:** Baseline characteristics of 2746 included patients, stratified by Mandard score

	All patients (*n* = 2746)	TRG1 (*n* = 608)	TRG2 (*n* = 526)	TRG3 (*n* = 565)	TRG4 (*n* = 389)	TRG5 (*n* = 182)	Partial regression (*n* = 476)
**Sex**							
Male	2317 (84.4)	506 (83.2)	441 (83.8)	470 (83.2)	340 (87.4)	154 (84.6)	406 (85.3)
Female	429 (15.6)	102 (16.8)	85 (16.2)	95 (16.8)	49 (12.6)	28 (15.4)	70 (14.7)
**Age (years), mean(s.d.)**	64.2(9.0)	64.6(9.3)	64.5(8.5)	64.4(8.7)	63.2(8.9)	64.2(9.6)	64.0(9.3)
**BMI (kg/m^2^), mean (s.d.)**	26.4(4.1)	26.4(4.1)	26.5(4.2)	26.5(4.2)	26.2(4.0)	26.3(4.4)	26.1(4.1)
**ASA fitness grade**							
I	516 (19.1)	109 (18.4)	89 (17.3)	106 (18.9)	85 (22.2)	28 (15.4)	99 (20.8)
II	1647 (60.8)	356 (60.1)	321 (62.3)	362 (64.5)	213 (55.6)	117 (64.3)	278 (58.5)
III–IV	545 (20.1)	127 (21.5)	105 (20.4)	93 (16.6)	85 (22.2)	37 (20.3)	98 (20.6)
**Clinical T category**							
cT1	33 (1.2)	11 (1.9)	7 (1.3)	7 (1.3)	3 (0.8)	1 (0.6)	4 (0.9)
cT2	503 (18.8)	136 (23.2)	96 (18.5)	97 (17.5)	60 (15.7)	33 (19.0)	81 (17.5)
cT3	2073 (77.4)	443 (73.9)	396 (76.3)	434 (78.2)	305 (79.8)	137 (78.8)	368 (79.7)
cT4	69 (2.6)	6 (1.0)	20 (3.9)	17 (3.1)	14 (3.7)	3 (1.7)	9 (1.9)
**Clinical N category**							
cN0	925 (34.1)	224 (37.5)	175 (33.5)	183 (32.4)	112 (29.3)	59 (32.8)	172 (36.9)
cN+	1786 (65.9)	373 (62.5)	347 (66.5)	381 (67.6)	270 (70.7)	121 (67.2)	294 (63.1)
**Tumour location**							
Distal oesophagus	2105 (76.7)	480 (78.9)	399 (75.9)	434 (76.8)	287 (73.8)	133 (73.1)	372 (78.2)
Gastro-oesophageal junction	641 (23.3)	128 (21.1)	127 (24.1)	131 (23.2)	102 (26.2)	49 (26.9)	104 (21.8)
**Procedure**							
Transhiatal	915 (33.3)	219 (36.0)	162 (30.8)	147 (26.0)	119 (30.6)	56 (30.8)	212 (44.5)
Transthoracic	1831 (66.7)	389 (64.0)	364 (69.2)	418 (74.0)	270 (69.4)	126 (69.2)	264 (55.5)
**Surgical approach**							
Open	1117 (40.8)	270 (44.4)	190 (36.2	194 (34.5)	139 (35.7)	65 (36.1)	259 (54.6)
Minimally invasive	1550 (56.6)	328 (53.9	321 (61.1)	345 (61.3)	238 (61.2)	109 (60.6)	209 (44.1)
Hybrid*	72 (2.6)	10 (1.6)	14 (2.7)	24 (4.3)	12 (3.1)	6 (3.3)	6 (1.3)
**Resection status**							
R0	2633 (96.1)	608 (100)	509 (97.0)	545 (96.8)	356 (91.5)	156 (85.7)	459 (96.8)
R+	108 (3.9)	0 (0)	16 (3.0)	18 (3.2)	33 (8.5)	26 (14.3)	15 (3.2)

Values are *n* (%). Owing to rounding, percentages may not add up to 100%. *Either thoracoscopy and laparotomy or thoracotomy and laparoscopy. TRG, tumour regression grade.

### Association between Mandard score and recurrence patterns

Recurrence rates increased with higher Mandard scores: 30.6% of patients in the TRG1 group developed recurrence, compared with 44.9, 52.9, 61.4, and 58.2% in the TRG2, TRG3, TRG4, and TRG5 groups respectively (*P* < 0.001). The higher the Mandard score, the higher the absolute number of both locoregional and distant recurrences (locoregional: TRG1 3.3%, TRG2 7.3%, TRG3 6.7%, TRG4 8.8%, TRG5 8.8%; distant: TRG1 20.2%, TRG2 27.7%, TRG3 33.2%, TRG4 39.4%, TRG5 38.7%; *P* < 0.001) (*Fig. 2c*).

**Fig. 3:**
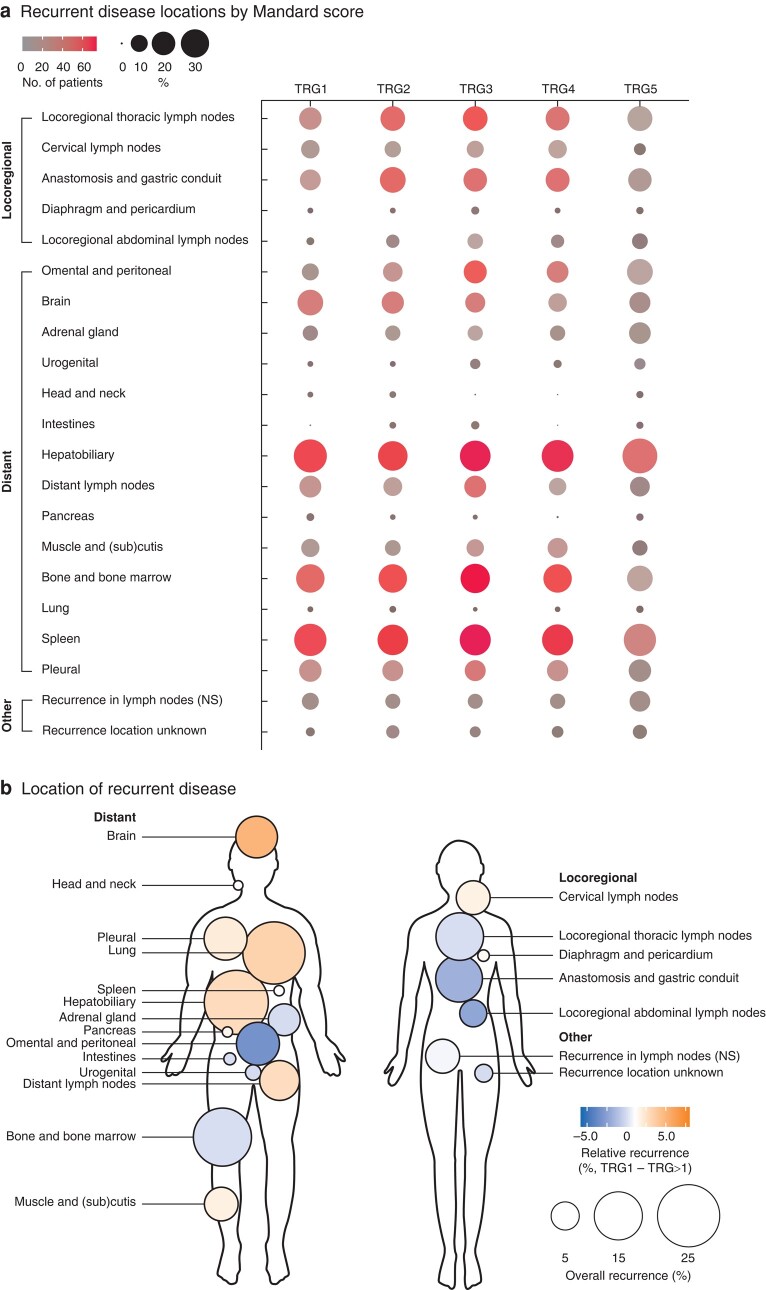
Specific locations of recurrent disease

Among patients with recurrent disease, the distribution of recurrence (locoregional *versus* distant) did not differ significantly between response groups (*P* = 0.797) (*Fig. 2d*). Comparison of recurrence location among patients with TRG1 *versus* those with partial or no regression (TRG>1) showed similar distribution (locoregional recurrence: 10.9% *versus* 14.1% respectively; distant recurrence: 66.8 *versus* 63.7%; combined: 22.3 *versus* 22.2%; *P* = 0.491) (*Fig. 2e*). Specific recurrence locations stratified by Mandard score are shown in *Fig. 3a* and *Table S1*. Patients with TRG1 were more often diagnosed with brain recurrences than those with TRG>1 (17.7 *versus* 9.8%; *P* = 0.001) and less often with omental/peritoneal (7.0 *versus* 12.8%; *P* = 0.025) and locoregional abdominal lymph node (1.1 *versus* 5.2%; *P* = 0.013) recurrences (*Fig. 3b* and*Table S2*).

**a** Specific locations of recurrent disease, stratified by Mandard score. The number of patients is indicated by the colour of the dot, and the recurrence rate by the size of the dot. **b** Specific locations of recurrent disease indicated by the size of the dot. By subtracting the recurrence rate for the group with TRG>1 from that for the group with TRG1, the relative recurrence rates were calculated to compare the recurrence rate in specific locations between the two groups. The relative percentage is indicated by the colour of the dot: red indicates a higher recurrence rate in the TRG1 group than in the TRG>1 group, and blue indicates a higher rate in the group with TRG>1. NS, not further specified.

**Fig. 4:**
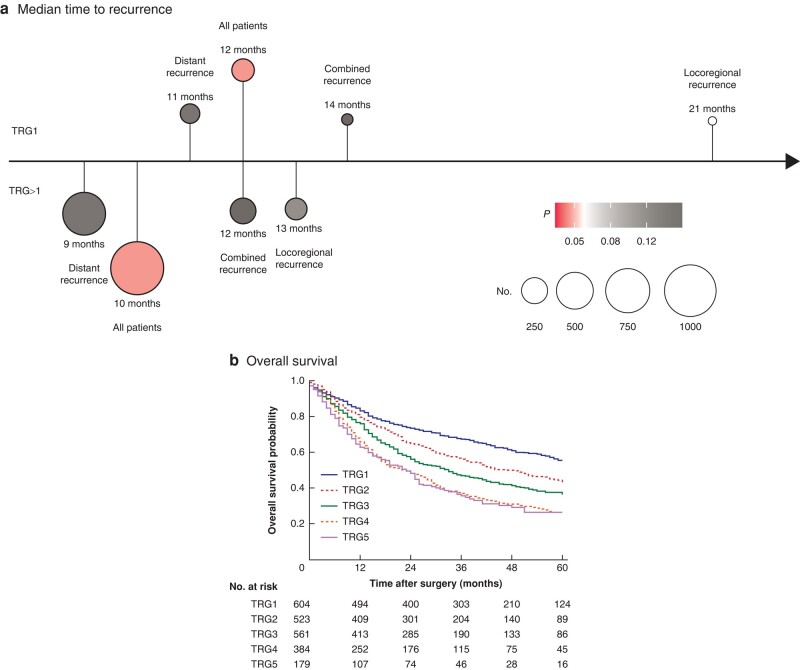
Time to recurrence and survival curves, stratified by Mandard score

The median time to recurrence was 12 (i.q.r. 10–14) months for patients with TRG1 compared with 10 (9–11) months for those with TRG>1 (*P* = 0.011) (*Fig. 4a*).

**a** Median time to recurrence among all patients and among patients with recurrence in different locations. The comparison is conducted between groups with TRG1 (top) and TRG>1 (bottom). The *P* value (log rank test) is indicated by the colour of the dot, and the number of patients by the size. **b** Overall survival among all patients, stratified by Mandard score; *P* < 0.001 (log rank test).

### Association between TRG-ypN score and recurrence patterns

**Fig. 5:**
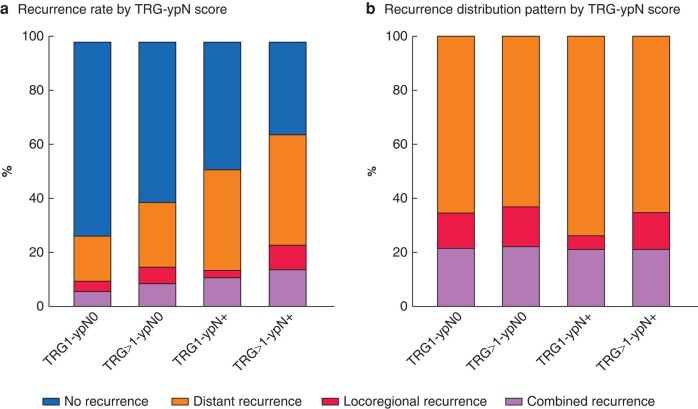
Recurrence patterns, stratified by TRG-ypN score

In the TRG-ypN score analyses, 529 patients (19.3%) were classified as having TRG1-ypN0, 1145 (41.7%) as TRG>1-ypN0, 79 (2.9%) as TRG1-ypN+, and 993 (36.2%) as TRG>1-ypN+ (*Fig. 1*). Recurrence rates differed significantly across the four categories, with the TRG>1-ypN+ group having the highest recurrence rate (64.9%) and the TRG1-ypN0 group the lowest (27.4%). The recurrence rate was higher for the TRG1-ypN+ group than for the TRG>1-ypN0 group (51.9 *versus* 39.6%; *P* < 0.001) (*Fig. 5a*).

**a** Recurrence rate by the modified response system among all 2746 patients with recorded Mandard score and pathological nodal status. **b** Recurrence distribution pattern by modified response system among 1272 patients with recurrence. **a***P* < 0.001, **b***P* = 0.719 (χ^2^ test).

**Fig. 6:**
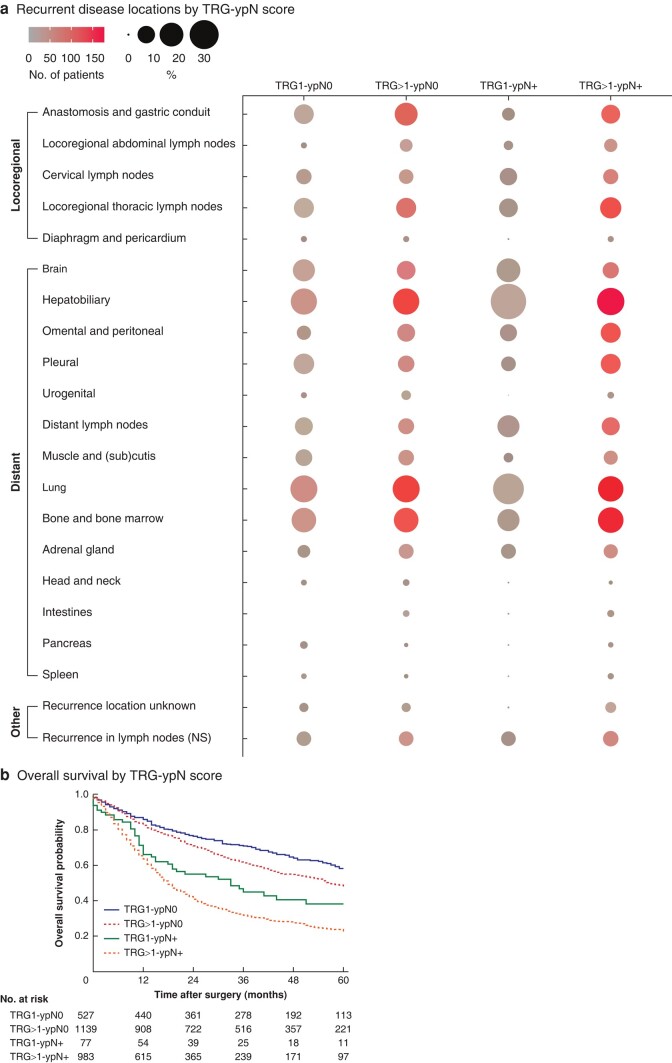
Impact of TRG-ypN score on patterns of recurrence and survival

Among patients with recurrence, the distribution (locoregional *versus* distant) did not differ between the four categories (*P* = 0.719) (*Fig. 5b*). However, the TRG1-ypN+ group tended towards fewer locoregional recurrences (4.9%) than the other groups (TRG1-ypN0 12.6%, TRG>1-ypN0 14.7%, TRG>1-ypN+ 13.6%), and more distant recurrences (73.2%) compared with the others (TRG1-ypN0 65.0%, TRG>1-ypN0 62.4%, TRG>1-ypN+ 64.6%). The results for specific recurrence sites in the four TRG-ypN groups are presented in *Fig. 6a* and *Table S3*. Recurrences at the anastomosis/gastric tube occurred significantly less often in the TRG1-ypN+ group compared with others (*P* = 0.007), although approximately half of the patients in the TRG1-ypN+ group with recurrent disease developed hepatobiliary metastases (46.3%), whereas this occurred less frequently in the other groups (24.1–27.3%) (*P* = 0.029). Recurrences in the brain occurred more frequently in the two TRG1 groups (TRG1-ypN0 17.2%, TRG1-ypN+ 19.5%) than in the two groups with TRG>1 (TRG>1-ypN0 11.7%, TRG>1-ypN+ 8.5%) (*P* = 0.004).

**a** Specific locations of recurrent disease, stratified by TRG-ypN score. The number of patients is indicated by the colour of the dot, and the recurrence rate by the size of the dot. **b** Overall survival, stratified by TRG-ypN score; *P* < 0.001 (log rank test). NS, not further specified.

### Correlation of both scoring systems with survival

Increasing Mandard scores were associated with progressively poorer prognosis (*Fig. 4b*). In patients with TRG1, mean OS was 44 (95% c.i. 42 to 45) months *versus* 35 (34 to 36) months for patients with TRG>1 (*P* < 0.001). This was also apparent in patients with recurrent disease, regardless of the location of recurrence. Among 173 patients with locoregional recurrence only, median OS was 46 (34 to 58) months for patients with TRG1 *versus* 25 (21 to 29) months for those with TRG>1 (*P* = 0.031). Among 716 patients with distant recurrence only, median OS was 19 (15 to 23) months and 15 (14 to 16) months respectively (*P* = 0.020) (*Fig. S1*).

Differences in survival between the four TRG-ypN score groups were observed (*P* < 0.001) (*Fig. 6b*); mean OS was best in patients with TRG1-ypN0 (45 (43 to 47) months), followed by TRG>1-ypN0 (41 (40 to 43) months), TRG1-ypN+ (33 (28 to 39) months), and TRG>1-ypN+ (27 (25 to 28) months).

## Discussion

The present study evaluated the prognostic value of the currently routinely applied Mandard score and the novel TRG-ypN score in relation to patterns of recurrent oesophageal adenocarcinoma and survival. It clearly demonstrated that, after neoadjuvant chemoradiotherapy and resection, isolated locoregional recurrence was uncommon. Patients with TRG1 developed recurrent disease less often than those with TRG>1 (31 *versus* 51%). Among patients with recurrent disease, the distribution of locoregional and distant recurrence was similar across the distinct response groups. TRG1 was associated with a higher incidence of tumour recurrence in the brain than TRG>1. In patients who had TRG1, the time to recurrence was 2 months longer and mean overall survival was 9 months longer than after an incomplete or absent response. The superior survival after TRG1 seems to contradict the recently published Neo-AEGIS trial^26^, in which, despite a higher frequency of TRG1, chemoradiotherapy failed to show a survival benefit compared with chemotherapy alone. It is hypothesized that this absence of a survival benefit for chemoradiotherapy in the Neo-AEGIS trial might have been due to both the low percentage of patients with TRG1 in the chemoradiotherapy group (TRG1 rate 12% *versus* 27% in the present study), as well as in the chemotherapy group (TRG1 rate 4%, reflecting an 8% difference), precluding a significant effect between groups on survival. It is also essential to recognize the nature of chemoradiotherapy as a predominantly local treatment, with limited systemic impact. Even when a pCR is achieved, the potential for distant metastases persists. Chemotherapy, on the other hand, operates more on a systemic level; even if it fails to yield a local pCR, it is capable of targeting cells responsible for distant metastases. Residual nodal disease was associated with a worse prognosis than residual disease at the primary tumour site in the oesophagus or gastro-oesophageal junction.

Intriguingly, it was shown that complete response to neoadjuvant chemoradiotherapy was associated with more recurrence in the brain, with an 8% difference in brain recurrence rate between TRG1 and TRG>1 groups (18 *versus* 10%). This finding is in line with previous research^27–29^. It is primarily hypothesized that the relatively long survival time of patients with TRG1 allows the detection of brain recurrence, leading to survivorship bias. Unfortunately, a record of the time to recurrence at specific sites is missing from the present data set. It is also possible that patients with TRG1 have specific molecular characteristics that make them more prone to developing brain metastases. The brain is a sanctuary recurrence site, owing to the presence of the blood–brain barrier (BBB) and the absence of cerebral lymphatic vessels. It could be hypothesized that chemoradiotherapy-sensitive tumours which responded completely had specific features that tended towards a different seeding pattern after treatment-induced epithelial–mesenchymal transition (EMT), increasing transmigration capability across the BBB and affinity towards the brain^30^. Studies^31,32^ involving patients with brain metastatic breast or lung cancer have provided evidence of EMT as an essential pattern of metastasis. International laboratory research evidence is necessary to ascertain the exact mechanism underlying brain recurrence subsequent to a pCR. Owing to the low incidence of brain recurrence, the number of patients who need to be screened in order to identify (and treat) cases of isolated brain recurrence early is exceptionally high. This means that active brain surveillance following treatment with curative intent for oesophageal cancer would require substantial resources, including time as well as financial investment, and is therefore not recommended based on the present results. A future international cohort study on recurrence patterns after neoadjuvant chemoradiotherapy and oesophagectomy is being planned with the TIGER study database, to further investigate the unexpected and somehow counterintuitive observation of more brain recurrences in patients with TRG1-ypN0^33^.

The current standard for assessing prognosis after the surgical removal of oesophageal malignancies is the eighth edition of the TNM classification^24^. Previous versions of this system were mainly based on patients who did not receive neoadjuvant therapy, and have proven to be less reliable in the prognostication of outcomes after chemoradiotherapy^34^. As a result, the eighth edition introduced a separate category for patients who have undergone neoadjuvant therapy (ypTNM). Despite this, the ypT category, whose definition is based on the largest depth of tumour invasion, may still not accurately foresee outcomes after neoadjuvant chemoradiotherapy owing to the unpredictable distribution of tumour cells in the oesophageal wall. To obviate this inadequacy, several systems have been developed that classify the histopathological response to neoadjuvant treatment^35^. Regression of the primary tumour might be more informative and may gain additional power to foresee outcomes in the postneoadjuvant treatment setting than ypT category^36,37^. Combining tumour regression at the primary tumour site with the pathological presence or absence of disease in the lymph nodes into a modified staging system probably has the potential to achieve improved prognostic accuracy including total tumour biology. Recently, Wong and colleagues^38^ demonstrated the prognostic superiority of a system including TRG and pN category over the use of ypT category for oesophageal squamous cell carcinoma. The present study is the first to describe the novel four-tier system for oesophageal adenocarcinoma, comparing complete responders *versus* non-complete responders: TRG1-ypN0, TRG>1-ypN0, TRG1-ypN+ *versus* TRG>1-ypN+. The primary reason for this subdivision and the decision not to consider the varying levels of residual disease was based on the superior outcome for patients with TRG1 compared with TRG>1, clearly setting them apart from the rest. Besides, a four-tier system is applicable and reproducible for prognostication of recurrence patterns and survival without too many complex groups, and overcomes the possible interpathologist variation in determining Mandard scores. On the contrary, selecting this subdivision led to the loss of nuanced distinctions between different response levels.

Patients in the TRG>1-ypN0 group developed fewer recurrences and had better survival than those in the TRG1-ypN+ group, which implies that residual nodal disease has a more negative prognostic impact than residual disease at the primary tumour site. In the TRG1-ypN+ group, adequate local tumour control is achieved as a result of a good response to neoadjuvant chemoradiotherapy, indicated by a recurrence rate at the anastomosis/gastric conduit of only 5%, *versus* 12–18% in the other groups. Furthermore, in the TRG1-ypN+ group, only 2.5% developed isolated locoregional recurrence, *versus* 5.8% of the TRG>1-ypN0 group. However, in contrast to the lower percentage of locoregional recurrence in the TRG1-ypN+ group, these patients developed 1.5 times as much distant recurrence as the group with TRG>1-ypN0 (38.0% *versus* 24.6%) and around half of the patients with TRG1-ypN+ who had recurrent disease developed brain and hepatobiliary metastases, associated with a poor prognosis^9^.

Pathological regression in the lymph nodes after neoadjuvant chemo(radio)therapy is a strong prognostic factor^19,39^. In the present study, the actual response of individual lymph nodes was unknown. Besides, it was decided not to consider pretreatment nodal status, because of the lack of reliability of clinical nodal staging in oesophageal cancer^40,41^. Furthermore, postneoadjuvant ypN category was previously shown to be more important than either pretreatment or change in nodal status^38^. Therefore, the authors chose to focus on pathological nodal status instead of lymph node response. For future research, it will be important to focus on the group of patients with inconsistency in response to therapy between the primary tumour site and the lymph nodes. It would also be interesting to elucidate the specific locations of clinically and pathologically positive nodes and relate these to the radiation field, as it has been shown that outfield metastases have poorer prognosis in oesophageal squamous cell carcinoma^42,43^.

Some limitations need to be considered when interpreting the present results. First, this was a retrospective analysis of prospectively collected data from multiple centres. There may have been intercentre variation in the management of oesophageal cancer (for example transthoracic and transhiatal, radical and non-radical oesophageal resections), which might be a confounding factor influencing recurrence patterns and survival. However, the multicentre approach allowed analysis of a large cohort of multimodally treated patients with oesophageal cancer in the Netherlands over a substantial time interval, notably highly representative of the current oesophageal cancer surgery practice. Second, for patients with multiple recurrence locations, only time to diagnosis of the first recurrence was recorded in the data set, and so the timing of subsequent recurrences was not taken into consideration. Besides, the exact number of recurrences was unknown, precluding comment on the prevalence of oligometastatic disease and curative local treatment options. It must also be acknowledged as a limitation that the ration fields were unknown, so it was not feasible to relate recurrence sites to inside or outside the radiation fields. Furthermore, some subanalyses had limited cohort sizes, which were of insufficient size for definitive conclusions to be drawn. Finally, the present results are only applicable to patients who received neoadjuvant chemoradiotherapy. Whether the score can be applied after neoadjuvant or perioperative chemotherapy needs to be investigated.

Although patients with a complete response developed less recurrence and had better outcomes, brain recurrences seemed to occur more frequently. This highlights the importance of comprehensive patient counselling. The prognosis was worse when residual cancer cells were present in the resected lymph nodes (TRG1-ypN+) than at the primary tumour site (TRG>1-ypN0). This information can guide the decision-making process regarding adjuvant treatment. The present findings have demonstrated that both TRG and ypN category are crucial components of any staging system, and neither alone can adequately prognosticate a patient’s outcome. As accurate prognostication is essential when communicating with the patient, as well as in the decision-making processes regarding adjuvant treatment and follow-up intensity, both factors should be considered.

## Collaborators

IVORY study group: Peter C. Baas, Renu R. Bahadoer, Eric J. T. Belt, Baukje Brattinga, Linda Claassen, Admira Ćosović, Manon Drost, Stijn van Esser, Marcia P. Gaspersz, Burak Görgec, Henk H. Hartgrink, Erwin van der Harst, Joos Heisterkamp, Wendy Kelder, B. Feike Kingma, Willem J. Koemans, Ewout A. Kouwenhoven, Frederik Lecot, Philip P. van der Linden, Grard A. P. Nieuwenhuijzen, Martijn G. H. van Oijen, Donald L. van der Peet, E. G. J. M. Robert Pierik, Fatih Polat, Rene Scheer, Cettela A. M. Slootmans, Odin V. Sosef, Wobbe O. de Steur, Hein B. A. C. Stockmann, Fanny J. Stoop, Guusje Vugts, Víola B. Weeda, Marinus J. Wiezer.

## Supplementary Material

znae034_Supplementary_Data

## Data Availability

Data from this study are not openly available, but are available on request with the permission of all authors and the IVORY study group.
