# Supplementary material for: Prognostic value of Mandard score and nodal status for recurrence patterns and survival after multimodal treatment of oesophageal adenocarcinoma
Source: Br J Surg. 2024 Feb 22;111(2):znae034. doi: 10.1093/bjs/znae034 (PMC10883709; doi:10.1093/bjs/znae034)
Supplement: znae034_Supplementary_Data [file znae034_supplementary_data.docx]

**TITLE**

**THE PROGNOSTIC VALUE OF MANDARD SCORE AND NODAL STATUS FOR RECURRENCE PATTERNS AND SURVIVAL AFTER MULTIMODAL TREATMENT OF ESOPHAGEAL ADENOCARCINOMA**

**AUTHORS**

Sofie PG Henckens^1-3^*, Dajia Liu^4,5^*, Suzanne S Gisbertz^1-3^, Marianne C Kalff^1-3^, Maarten CJ Anderegg^1-3^, David Crull^6^, Freek Daams^7^, Annette D van Dalsen^8^, Jan Willem T Dekker^9^, Marc J van Det^6^, Peter van Duijvendijk^10^, Wietse J Eshuis^1-3^, Richard PR Groenendijk^11^, Jan Willem Haveman^12^, Richard van Hillegersberg^13^, Misha DP Luyer^14^, Pim B Olthof^9^, Jean-Pierre EN Pierie^15^, Victor D Plat^7^, Camiel Rosman^16^, Jelle P Ruurda^13^, Johanna W van Sandick^17^, Meindert N Sosef^18^, Daan M Voeten^1-3^, Guy HEJ Vijgen^19^, Maarten F Bijlsma^2,5,20^, Sybren L Meijer^2,21^, Maarten CCM Hulshof^2,22^, Cesar Oyarce^5,20^, Sjoerd M Lagarde^19^, Hanneke WM van Laarhoven^2,4,†^, Mark I van Berge Henegouwen^1-3†^, *on behalf of the IVORY study group*

**AFFILIATIONS**

1. Amsterdam UMC, location University of Amsterdam, Department of Surgery, Meibergdreef 9, Amsterdam, the Netherlands
2. Cancer Center Amsterdam, Cancer Treatment and Quality of Life, Amsterdam, the Netherlands
3. Amsterdam UMC, location University of Amsterdam, Department of Gastroenterology and Hepatology, Amsterdam Gastroenterology Endocrinology Metabolism, Meibergdreef 9, Amsterdam, the Netherlands
4. Amsterdam UMC, location University of Amsterdam, Department of Medical Oncology, Meibergdreef 9, Amsterdam, the Netherlands
5. Amsterdam UMC, location University of Amsterdam, Center for Experimental and Molecular Medicine, Laboratory for Experimental Oncology and Radiobiology, Amsterdam, the Netherlands
6. Ziekenhuisgroep Twente, Department of Surgery, Almelo, the Netherlands
7. Amsterdam UMC, location Vrije Universiteit Amsterdam, Department of Surgery, De Boelelaan 1117, Amsterdam, the Netherlands
8. Isala Klinieken, Department of Surgery, Zwolle, the Netherlands
9. Reinier de Graaf Groep, Department of Surgery, Delft, the Netherlands
10. Gelre Ziekenhuis, Department of Surgery, Apeldoorn, the Netherlands
11. IJsselland Ziekenhuis, Department of Surgery, Capelle aan den IJssel, the Netherlands
12. University Medical Centre Groningen, University of Groningen, Department of Surgery, Groningen, the Netherlands
13. UMC Utrecht, Department of Surgery, Utrecht, the Netherlands
14. Catharina Ziekenhuis, Department of Surgery, Eindhoven, the Netherlands
15. MC Leeuwarden, Department of Surgery, Leeuwarden, the Netherlands
16. Radboud University Medical Center, Department of Surgery, Nijmegen, the Netherlands
17. Antoni van Leeuwenhoek Ziekenhuis, Department of Surgery, Amsterdam, the Netherlands
18. Zuyderland, Department of Surgery, Heerlen, the Netherlands
19. Erasmus Medical Center, Department of Surgery, Rotterdam, the Netherlands
20. Oncode Institute, Amsterdam, the Netherlands
21. Amsterdam UMC, location University of Amsterdam, Department of Pathology, Meibergdreef 9, Amsterdam, the Netherlands
22. Amsterdam UMC, location University of Amsterdam, Department of Radiotherapy, Meibergdreef 9, Amsterdam, the Netherlands

^*^ These authors share first authorship, both authors contributed equally to this article

^†^ These authors share senior authorship

**CORRESPONDING AUTHOR**

Prof. dr. M. I. van Berge Henegouwen

Amsterdam UMC, location University of Amsterdam, Department of Surgery

Meibergdreef 9, 1105 AZ Amsterdam, The Netherlands

Tel: +31 205669111

Mail: m.i.vanbergehenegouwen@amsterdamumc.nl

**Supplementary Materials - Index**

| **Supplementary Figures and Tables** |  |
| --- | --- |
| Supplementary Table 1. | *pag. 3* |
| Supplementary Table 2. | *pag. 4* |
| Supplementary Table 3. | *pag. 5* |
| Supplementary Figure 1. | *pag. 6* |

**Supplementary Figures and Tables**

**Supplementary Table 1.** Specific recurrence locations, stratified by Mandard score

| **Site of recurrence** | **All** | | **TRG1** | | **TRG2** | | **TRG3** | | **TRG4** | | **TRG5** | | **p value** |
| --- | --- | --- | --- | --- | --- | --- | --- | --- | --- | --- | --- | --- | --- |
|  | **n** | **%** | **n** | **%** | **n** | **%** | **n** | **%** | **n** | **%** | **n** | **%** |  |
| **Locoregional** |  |  |  |  |  |  |  |  |  |  |  |  |  |
| Locoregional abdominal lymph nodes | 46 | 4.4 | 2 | 1.1 | 10 | 4.3 | 18 | 6.1 | 10 | 4.2 | 6 | 5.7 | 0.122 |
| Cervical lymph nodes | 73 | 6.9 | 15 | 8.2 | 16 | 6.8 | 20 | 6.7 | 19 | 8.1 | 3 | 2.9 | 0.461 |
| Anastomosis and gastric conduit | 151 | 14.3 | 21 | 11.4 | 39 | 16.7 | 41 | 13.8 | 35 | 14.8 | 15 | 14.3 | 0.656 |
| Diaphragm and pericardium | 8 | 0.8 | 1 | 0.5 | 1 | 0.4 | 4 | 1.3 | 1 | 0.4 | 1 | 1.0 | n/a |
| Locoregional thoracic lymph nodes | 160 | 15.2 | 25 | 13.6 | 38 | 16.2 | 46 | 15.5 | 34 | 14.4 | 17 | 16.2 | 0.941 |
| **Distant** |  |  |  |  |  |  |  |  |  |  |  |  |  |
| Omental and peritoneal | 127 | 12.0 | 13 | 7.1 | 23 | 9.8 | 44 | 14.8 | 29 | 12.3 | 18 | 17.1 | **0.038** |
| Brain | 125 | 11.8 | 33 | 17.9 | 29 | 12.4 | 31 | 10.4 | 20 | 8.5 | 12 | 11.4 | **0.045** |
| Adrenal gland | 67 | 6.3 | 10 | 5.4 | 14 | 6.0 | 18 | 6.1 | 12 | 5.1 | 13 | 12.4 | 0.115 |
| Urogenital | 15 | 1.4 | 1 | 0.5 | 1 | 0.4 | 7 | 2.4 | 3 | 1.3 | 3 | 2.9 | n/a |
| Intestines | 7 | 0.7 | 0 | 0.0 | 2 | 0.9 | 4 | 1.3 | 0 | 0.0 | 1 | 1.0 | n/a |
| Hepatobiliary | 282 | 26.7 | 54 | 29.3 | 55 | 23.5 | 73 | 24.6 | 65 | 27.5 | 35 | 33.3 | 0.286 |
| Head and neck | 4 | 0.4 | 1 | 0.5 | 2 | 0.9 | 0 | 0.0 | 0 | 0.0 | 1 | 1.0 | n/a |
| Distant lymph nodes | 106 | 10.0 | 23 | 12.5 | 20 | 8.5 | 35 | 11.8 | 18 | 7.6 | 10 | 9.5 | 0.359 |
| Muscle and (sub)cutis | 81 | 7.7 | 15 | 8.2 | 14 | 6.0 | 22 | 7.4 | 24 | 10.2 | 6 | 5.7 | 0.451 |
| Pancreas | 5 | 0.5 | 2 | 1.1 | 1 | 0.4 | 1 | 0.3 | 0 | 0.0 | 1 | 1.0 | n/a |
| Bone and bone marrow | 228 | 21.6 | 39 | 21.2 | 50 | 21.4 | 70 | 23.6 | 50 | 21.2 | 19 | 18.1 | 0.828 |
| Lung | 272 | 25.8 | 52 | 28.3 | 57 | 24.4 | 74 | 24.9 | 60 | 25.4 | 29 | 27.6 | 0.885 |
| Spleen | 6 | 0.6 | 1 | 0.5 | 2 | 0.9 | 1 | 0.3 | 1 | 0.4 | 1 | 1.0 | n/a |
| Pleural | 120 | 11.4 | 24 | 13.0 | 26 | 11.1 | 32 | 10.8 | 25 | 10.6 | 13 | 12.4 | 0.927 |
| **Other** |  |  |  |  |  |  |  |  |  |  |  |  |  |
| Recurrence in lymph nodes (NS) | 66 | 6.3 | 13 | 7.1 | 13 | 5.6 | 16 | 5.4 | 13 | 5.5 | 11 | 10.5 | 0.380 |
| Recurrence location unknown | 25 | 2.4 | 2 | 1.1 | 8 | 3.4 | 6 | 2.0 | 5 | 2.1 | 4 | 3.8 | 0.465 |

***Abbreviations***:

NS = Not further Specified
n/a = not applicable, groups too small for comparison

**Supplementary Table 2.** Specific recurrence locations, stratified by regression status (TRG1 vs. TRG>1)

| **Site of recurrence** | **All** | | **TRG1** | | **TRG>1** | | **p value** |
| --- | --- | --- | --- | --- | --- | --- | --- |
|  | **n** | **%** | **n** | **%** | **n** | **%** |  |
| **Locoregional** |  |  |  |  |  |  |  |
| Locoregional abdominal lymph nodes | 59 | 4.6 | 2 | 1.1 | 57 | 5.2 | **0.013** |
| Anastomosis and gastric conduit | 181 | 14.2 | 21 | 11.4 | 160 | 14.7 | 0.237 |
| Cervical lymph nodes | 94 | 7.4 | 15 | 8.2 | 79 | 7.3 | 0.669 |
| Diaphragm and pericardium | 10 | 0.8 | 1 | 0.5 | 9 | 0.8 | n/a |
| Locoregional thoracic lymph nodes | 184 | 14.5 | 25 | 13.6 | 159 | 14.6 | 0.714 |
| **Distant** |  |  |  |  |  |  |  |
| Brain | 141 | 11.1 | 33 | 17.9 | 108 | 9.9 | **0.001** |
| Omental and peritoneal | 153 | 12.0 | 13 | 7.1 | 140 | 12.9 | **0.025** |
| Intestines | 11 | 0.9 | 0 | 0.0 | 11 | 1.0 | n/a |
| Urogenital | 19 | 1.5 | 1 | 0.5 | 18 | 1.7 | 0.251 |
| Distant lymph nodes | 129 | 10.1 | 23 | 12.5 | 106 | 9.7 | 0.252 |
| Lung | 317 | 24.9 | 52 | 28.3 | 265 | 24.4 | 0.257 |
| Pancreas | 7 | 0.6 | 2 | 1.1 | 5 | 0.5 | n/a |
| Hepatobiliary | 340 | 26.7 | 54 | 29.3 | 286 | 26.3 | 0.386 |
| Pleural | 150 | 11.8 | 24 | 13.0 | 126 | 11.6 | 0.569 |
| Adrenal gland | 81 | 6.4 | 10 | 5.4 | 71 | 6.5 | 0.575 |
| Muscle and (sub)cutis | 93 | 7.3 | 15 | 8.2 | 78 | 7.2 | 0.636 |
| Bone and bone marrow | 280 | 22 | 39 | 21.2 | 241 | 22.2 | 0.772 |
| Head and neck | 6 | 0.5 | 1 | 0.5 | 5 | 0.5 | n/a |
| Spleen | 7 | 0.6 | 1 | 0.5 | 6 | 0.6 | n/a |
| **Other** |  |  |  |  |  |  |  |
| Recurrence location unknown | 26 | 2.0 | 2 | 1.1 | 24 | 2.2 | 0.321 |
| Recurrence in lymph nodes (NS) | 94 | 7.4 | 13 | 7.1 | 81 | 7.4 | 0.856 |

***Abbreviations***:

NS = Not further Specified
n/a = not applicable, groups too small for comparison

**Supplementary Table 3.** Specific recurrence locations, stratified by modified response system (TRG1-ypN0 vs. TRG>1-ypN0 vs. TRG1-ypN+ vs. TRG>1-ypN+)

| **Site of recurrence** | **All** | | **TRG1-ypN0** | | **TRG>1-ypN0** | | **TRG1-ypN+** | | **TRG>1-ypN+** | | **p value** |
| --- | --- | --- | --- | --- | --- | --- | --- | --- | --- | --- | --- |
|  | **n** | **%** | **n** | **%** | **n** | **%** | **n** | **%** | **n** | **%** |  |
| **Locoregional** |  |  |  |  |  |  |  |  |  |  |  |
| Anastomosis and gastric conduit | 181 | 14.1 | 19 | 13.1 | 83 | 18.3 | 2 | 4.9 | 77 | 12.0 | **0.007** |
| Locoregional abdominal lymph nodes | 59 | 4.6 | 1 | 0.7 | 22 | 4.9 | 1 | 2.4 | 35 | 5.4 | 0.051 |
| Cervical lymph nodes | 94 | 7.3 | 11 | 7.6 | 30 | 6.6 | 4 | 9.8 | 49 | 7.6 | 0.785 |
| Locoregional thoracic lymph nodes | 184 | 14.3 | 20 | 13.8 | 61 | 13.5 | 5 | 12.2 | 98 | 15.2 | 0.836 |
| Diaphragm and pericardium | 10 | 0.8 | 1 | 0.7 | 4 | 0.9 | 0 | 0.0 | 5 | 0.8 | n/a |
| **Distant** |  |  |  |  |  |  |  |  |  |  |  |
| Brain | 141 | 11.0 | 25 | 17.2 | 53 | 11.7 | 8 | 19.5 | 55 | 8.5 | **0.004** |
| Hepatobiliary | 340 | 26.5 | 35 | 24.1 | 110 | 24.3 | 19 | 46.3 | 176 | 27.3 | **0.018** |
| Omental and peritoneal | 153 | 11.9 | 9 | 6.2 | 48 | 10.6 | 4 | 9.8 | 92 | 14.3 | **0.029** |
| Pleural | 150 | 11.7 | 21 | 14.5 | 41 | 9.1 | 3 | 7.3 | 85 | 13.2 | 0.096 |
| Urogenital | 19 | 1.5 | 1 | 0.7 | 12 | 2.6 | 0 | 0.0 | 6 | 0.9 | 0.121 |
| Distant lymph nodes | 129 | 10.1 | 16 | 11.0 | 37 | 8.2 | 7 | 17.1 | 69 | 10.7 | 0.189 |
| Muscle and (sub)cutis | 93 | 7.2 | 14 | 9.7 | 36 | 7.9 | 1 | 2.4 | 42 | 6.5 | 0.354 |
| Lung | 317 | 24.7 | 38 | 26.2 | 114 | 25.2 | 14 | 34.1 | 151 | 23.4 | 0.435 |
| Bone and bone marrow | 280 | 21.8 | 32 | 22.1 | 94 | 20.8 | 7 | 17.1 | 147 | 22.8 | 0.749 |
| Adrenal gland | 81 | 6.3 | 7 | 4.8 | 31 | 6.8 | 3 | 7.3 | 40 | 6.2 | 0.823 |
| Head and neck | 6 | 0.5 | 1 | 0.7 | 4 | 0.9 | 0 | 0.0 | 1 | 0.2 | n/a |
| Intestines | 11 | 0.9 | 0 | 0.0 | 4 | 0.9 | 4 | 0.0 | 7 | 1.1 | n/a |
| Pancreas | 7 | 0.5 | 2 | 1.4 | 1 | 0.2 | 0 | 0.0 | 4 | 0.6 | n/a |
| Spleen | 7 | 0.5 | 1 | 0.7 | 1 | 0.2 | 0 | 0.0 | 5 | 0.8 | n/a |
| **Other** |  |  |  |  |  |  |  |  |  |  |  |
| Recurrence location unknown | 36 | 2.8 | 3 | 2.1 | 10 | 2.2 | 0 | 0.0 | 23 | 3.6 | 0.460 |
| Recurrence in lymph nodes (NS) | 94 | 7.3 | 10 | 6.9 | 33 | 7.3 | 3 | 7.3 | 48 | 7.5 | n/a |

***Abbreviations***:

NS = Not further Specified
n/a = not applicable, groups too small for comparison

**
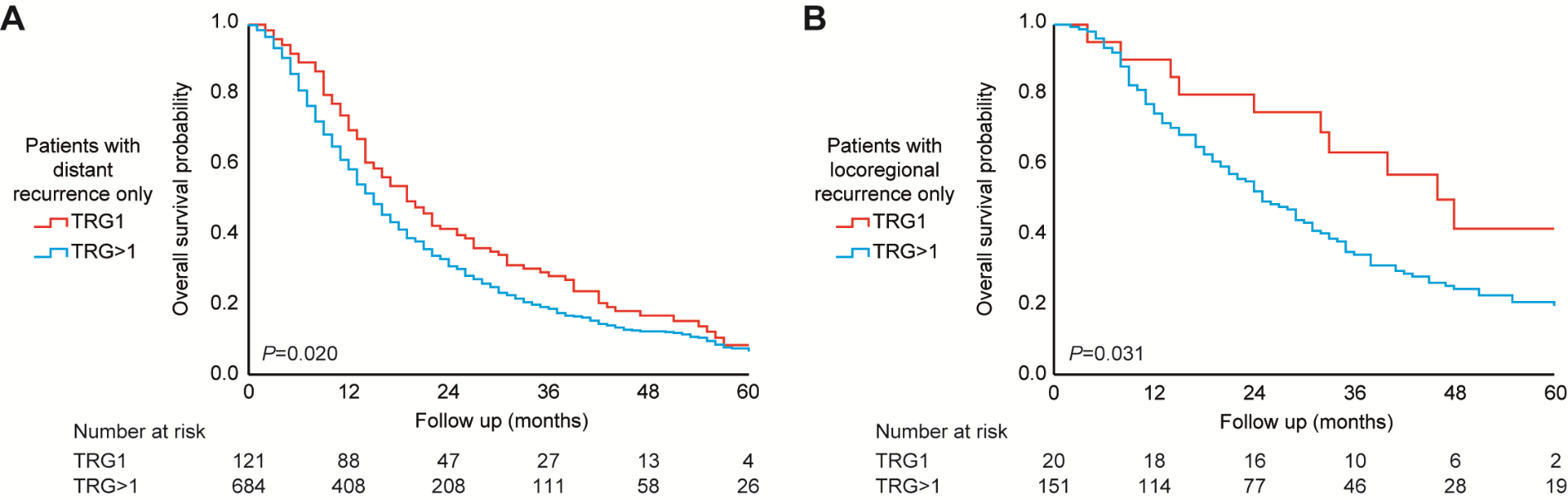
**

**
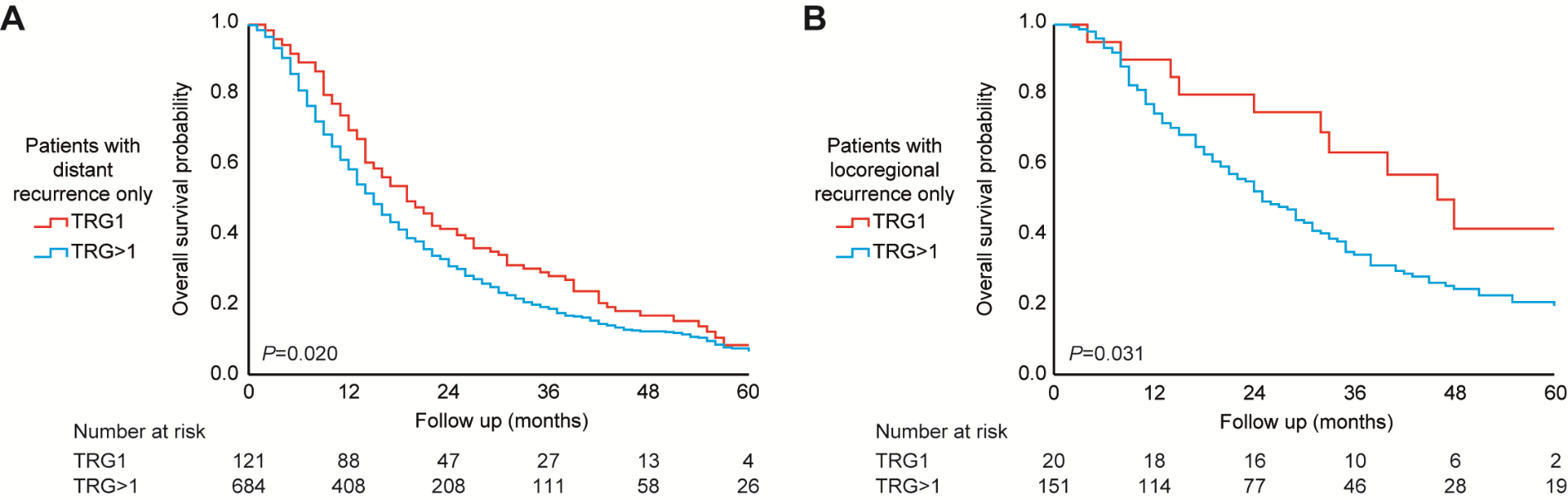
**

**Supplementary Figure 1.** Overall survival curves apart for patients with distant and locoregional recurrence, stratified by regression status

**A)** Overall survival in patients with distant recurrence only, stratified by regression status (TRG1 vs. TRG>1)

**B)** Overall survival in patients with locoregional recurrence only, stratified by regression status (TRG1 vs. TRG>1
